# A Review with a Focus on *Vaccinium*-Berries-Derived Bioactive Compounds for the Treatment of Reproductive Cancers

**DOI:** 10.3390/plants13071047

**Published:** 2024-04-08

**Authors:** Naser A. Alsharairi

**Affiliations:** Heart, Mind and Body Research Group, Griffith University, Gold Coast, QLD 4222, Australia; naser.alsharairi@gmail.com

**Keywords:** *Vaccinium* berries, bioactive compounds, molecular mechanisms, prostate cancer, bladder cancer, ovarian cancer, cervical cancer

## Abstract

Cancers of the reproductive organs, including prostate, bladder, ovarian, and cervical cancers, are considered the most common causes of death in both sexes worldwide. The genus *Vaccinium* L. (Ericaceae) comprises fleshy berry crop species, including cranberries, blueberries, lingonberries, bilberries, and bog bilberries, and are widely distributed in many countries. Flavonols, anthocyanins (ACNs), proanthocyanidins (PACs), and phenolic acids are the most bioactive compounds naturally found in *Vaccinium* berries and have been extensively used as anticancer agents. However, it remains uncertain whether *Vaccinium* bioactives have a therapeutic role in reproductive cancers (RCs), and how these bioactives could be effective in modulating RC-related signalling pathways/molecular genes. Therefore, this article aims to review existing evidence in the PubMed/MEDLINE database on *Vaccinium* berries’ major bioactive compounds in RC treatment and unravel the mechanisms underlying this process.

## 1. Introduction

Reproductive cancers (RCs), including prostate, cervix uteri, vagina, and vulva cancers have a large impact on cancer deaths, particularly in transitioning countries across Asia, Africa, and South America [1]. Prostate and bladder cancers are the most common male genital malignancies. Worldwide, prostate cancer (PCa) is the second most common cancer in men aged ≥50 years, accounting for 1,414,259 cases in 2020 [2]. The PCa death rate is expected to be 7.5 million in 2040, a double increase from 2020 [3]. Bladder cancer (BC) incidences and death rates are four times higher in men than in women. There were an estimated 573,000 new cases and 213,000 deaths due to BC in 2020. The numbers of BC cases and deaths are anticipated to increase to 991,000 and 397,000, respectively, by 2040 [4]. Occupational exposures and tobacco smoking are the major risk factors for PCa and BC. Many other factors have also been associated with an increased risk of these cancers, such as genetic predisposition, family history, older age, low physical activity, coffee intake, high vitamin B1 intake, low fruits, and vegetables intake, low serum vitamin D levels, imbalanced microbiome, specific medications, and pelvic radiotherapy [5,6].

Ovarian and cervical cancers are regarded as the most common gynaecologic malignancies, whereas vaginal, uterine, and vulvar cancers are relatively rare [7]. Ovarian cancer (OC) is the eighth most common cancer in women worldwide, with 314,000 cases and 207,000 deaths reported in 2020; these numbers are projected to double in 2040 [8]. The aetiology of OC is attributed to several risk factors, such as coffee and fat intake, hormone therapies (e.g., oestrogen, oestrogen–progesterone, and menopausal hormone use), genetic factors, smoking, obesity, and diabetes [9]. Globally, cervical cancer (CC) constitutes the third most common cancer in women, with 604,127 cases and 341,831 deaths estimated in 2020 [10]. Long-term use of oral contraceptive pills, sexually transmitted agents, multiple sexual partners, and tobacco smoking have been recognized as the major risk factors for CC [11].

Fruits can be classified into dry and fleshy types based on their water content at ripening [12]. Fleshy fruits are characterized by high water content and are grown in habitats at relatively low altitudes [12]. Fleshy-fruited species undergo stages of development known as fruit set, initiation, growth, maturation, and finally ripening [13]. Fleshy fruits consist of pericarp tissue layers originating from the ovary wall of a flowering plant and tissues other than the ovary [12]. Fleshy fruit pericarps play an important role in dispersing and nurturing seeds [12]. The pericarp is typically divided into three distinct layers: the endocarp (inner layer), the mesocarp (intermediate layer), and the exocarp (outer skin). The exocarp supports internal cell layers and aids in the evolution of fleshy fruits with soft edible tissue [12]. Fleshy fruits can be further categorized into climacteric and non-climacteric fruits based on their ethylene and abscisic acid (ABA) production [13]. Ethylene and ABA engage in crosstalk with phytohormones (e.g., auxin, cytokinin, brassinosteroids, and gibberellins) to regulate fertilization and pollination [13,14]. 

*Vaccinium* berries are fleshy fruits and are classified as non-climacteric fruits based on their ripening process [15]. The genus *Vaccinium*, which belongs to the family Ericaceae, comprises a large group of berry crop species that are widely distributed in North and Central America, Africa, Asia, and Europe [15]. The most popular species in this genus are cranberries (*V. macrocarpon* L./*oxycoccos* L.), bilberries (*V. myrtillus* L.), bog bilberries (*V. uliginosum* L.), lingonberries (*V. vitis-idaea* L.), and blueberries (*V. corymbosum* L./*angustifolium* L.) [15,16]. The profile of *Vaccinium* berries is rich in phenolic compounds such as anthocyanins (ACNs), proanthocyanidins (PACs), and flavonols [17]. The contents and levels of *Vaccinium* bioactives are considerably influenced by species, cultivation conditions, latitude, and different stages of ripening [18]. Flavonols, PACs, and hydroxinnamic acids are the main phenolic compounds present during berry development, whereas ACNs are synthesised and accumulate during early stages of ripening [19]. ACNs are responsible for the vivid colours that are used as signals to attract seed dispersers and pollinators [19]. The PACs profile of cranberries, lingonberries, bog bilberries, and bilberries consists of procyanidins and is high in A-type trimers and dimmers [20,21]. In *V. myrtillus* L., ACN accumulation in fruit skin and flesh is a key indicator of fruit ripening [22]. Both endogenous and exogenous ABA induces ABA biosynthesis-related gene expression, leading to increased ACN accumulation, which gives the fruit its blue colour [23,24]. Increasing accumulation of dark blue ACN pigments (particularly petunidin glycoside, delphinidin, and malvidin) in the skin of the highbush blueberry (*V. corymbosum* L.) corresponds to the increase in ABA biosynthesis at ripening initiation [25]. *V. uliginosum* L. accumulates high levels of five dark blue to black ACN pigments (petunidin, cyanidin, delphinidin, malvidin, and peonidin) in its skin, with malvidin glucosides being the most abundant [26]. The profiles of red ACNs accumulated in the skins of *V. macrocarpon* L. and *V. oxycoccos* L. consist of six pigments, with peonidin-3-galactoside being the major one [27]. 

Understanding the molecular classifications of RCs could provide directions for successful targeted therapies. The common treatment options for male and female RCs are chemotherapy, immunotherapy, and radiotherapy [28,29,30,31]. PCa is classified into several subtypes with different clinicopathological features based on eight molecular classifications: tomlins, the cancer genome atlas (TCGA), prostate cancer 14-pathway (PCS), 50-gene signature (PAM50), bone-metastatic, epithelial, immune, and multi-omics [28]. The proliferation and progression of PCa cells are dependent on androgen-induced androgen receptor (AR) signalling, which is associated with high activation of oncogenic signalling pathways such as signal transducer and activator of transcription 3 (STAT3) [28]. The PCS, PAM50, epithelial, and multi-omics classifications are likely to represent the luminal A subtype, which is characterized by high activity of AR, sensitivity to androgen deprivation therapy, and elevated proliferation genes such as forkhead box A1 (FOXA1) and hypoxia-inducible factor-1α (HIF1α) [28]. BC is classified into basal and luminal subtypes based on gene expression profiles and different molecular classification systems. These subtypes exhibit features of neuroendocrine/squamous differentiation, fibroblast, papillary histology, immune infiltration, peroxisome proliferator-activated receptor gamma (PPARγ) signature, mutations in fibroblast growth factor receptor 3 (FGFR3), lysine (K)-specific demethylase 6 (KDM6), p53 tumour-suppressor gene (TP53), and retinoblastoma gene 1 (RB1) [29]. OC consists of five main histologic subtypes: high-grade serous, low-grade serous, clear cell, endometrioid, and mucinous carcinoma. These subtypes share chromosomal instability features and represent different clinical characteristics, mutated genes, aggressive clinical course, and immunohistochemical markers (Wilms’ tumour gene 1, WT1; protein 53, p53; progesterone receptor, PR; napsin A) [30]. Currently, three aggressive CC subtypes have been identified: gastric-type adenocarcinoma, carcinosarcoma, and small-cell carcinoma of the cervix (SCCC). SCCC and gastric-type adenocarcinoma are characterized by high genetic alterations, including mutations in TP53, Kirsten rat sarcoma viral oncogene homologue (KRAS), phosphatase and tensin homologue (PTEN), AT-rich interaction domain 1A (ARID1A), and p110alpha catalytic subunit of PI3K (PIK3CA). Immune checkpoint inhibitors such as poly(ADP-ribose) polymerase (PARP) inhibitors have shown the potential to inhibit PARP expression in SCCC cells [31]. 

A recent review of experimental studies has demonstrated that *Vaccinium* bioactives, including ACNs, PACs, gallic acid, and hippuric acid, have therapeutic potential in breast cancer treatment. These bioactives exert antiproliferative, anti-inflammatory, apoptotic, and autophagic activities against breast cancer cells through inhibition and/or activation of molecular genes as potential mechanisms for these effects [32]. Despite a few preclinical studies showing results of cranberry PACs in PCa and OC treatment [33], to date, no review has provided a comprehensive understanding of the role of *Vaccinium* bioactives in RC treatment. Thus, this review aims to summarize experimental studies and/or clinical trials on *Vaccinium* berries’ major bioactives in RC treatment and to unravel the molecular mechanisms underlying their roles in the treatment.

## 2. Methods

A literature search of the PubMed/Medline database up to February 2024 was performed. All articles related to *Vaccinium*-berries-derived bioactive compounds in RC treatment published in English were considered. The search terms used to retrieve articles were: *Vaccinium* berries, bilberry, lingonberry, bog bilberry, bearberry, cranberry, blueberry, PCa, BC, OC, CC, testicular cancer, vaginal cancer, endometrial cancer, uterine cancer, and vulvar cancer. The search terms were combined using the Boolean operator ‘AND’. The titles and abstracts of the studies were evaluated based on the search terms. A total of 34 articles were identified and selected for inclusion from an initial 119 articles. 

## 3. *Vaccinium* Berries as Sources of Bioactive Compounds

**Figure 1:**
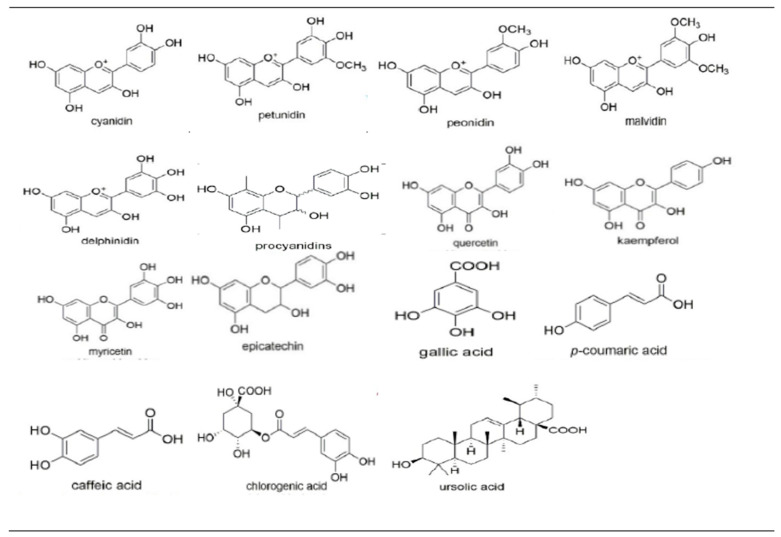
Phytochemical composition of *Vaccinium* berries with anticancer properties against RC cells [16].

The most bioactive compounds extracted from *Vaccinium* spp. (blueberry, cranberry, bilberry, and lingonberry) are ACNs (cyanidin, peonidin, petunidin, delphinidin, malvidin), PACs, flavonols (quercetin; Qu, kaempferol; Km, myricetin; Myr), flavanols (epicatechin), phenolic acids (gallic acid, p-coumaric acid, caffeic acid, chlorogenic acid), and ursolic acid. Each bioactive compound has a unique chemical structure [16] that may exert anticancer effects on RC cells. The phytochemical composition of these bioactives is presented in Figure 1. 

## 4. The Therapeutic Role of Blueberry Bioactives in Reproductive Cancers

The therapeutic effects of blueberry bioactives against RC cells have been evaluated in a few experiments, together with their role in inhibiting proliferation, adhesion, viability, inflammation, mutagenesis, and metastasis and inducing apoptosis and cell cycle arrest. One in vitro experiment showed that highbush blueberry (*V. corymbosum*), lowbush blueberry (*V. angustifolium*), and velvet leaf blueberry (*V. myrtilloides*) inhibit the proliferation of PCa cells at different concentrations. This inhibition was mediated by inducing cell cycle arrest at the G_1_ phase by downregulating the expression of multiple genes associated with the cell cycle [34]. Velvet leaf blueberry was also reported to inhibit inflammation in PCa cells by downregulating tumour necrosis factor-α (TNFα)-induced cyclooxygenase-2 (COX-2) and nuclear factor kappa-B (NFkB) expression [34]. The results of the in vitro experiment showed that treatment with three flavonoid-enriched fractions (a crude extract that contains all flavonoids, ACNs, and PACs) from lowbush blueberries inhibits PCa metastasis at concentrations of 0.5–1.0 mg/mL by decreasing the expression of matrix metalloproteinases (MMPs) [35]. The experiment also demonstrated necrotic/apoptotic cell death at the same concentrations through the activation of caspase-3 expression. However, the reduction in MMP expression observed in PCa cells in response to treatment was not due to necrotic/apoptotic cell death [35]. The same flavonoid-enriched fractions from lowbush blueberries also inhibited MMP activity, while increasing metalloproteinase (TIMP) activity when tested in another in vitro experiment. The mechanisms underlying these actions are associated with the inhibition of protein kinase A (PKA) and mitogen-activated protein (MAP) kinase signalling pathways [36]. 

Blueberries were also shown to inhibit the proliferation and/or adhesion of PC cells, but the mechanisms underlying these activities have not been elucidated. Treatment with PAC-enriched fractions from wild blueberry fruits (*V. angustifolium* Ait.) (fractions four and five) at a concentration of 20 μg/mL and 2.38 mM QU standard resulted in the inhibition of PCa cell proliferation and adhesion in vitro, with low toxic effects being observed. These fractions contained 4 → 8-linked oligomeric PACs with degrees of polymerization (DPn) of 3.25 and 5.65, which resulted in high anti-proliferation and anti-adhesion activities in PCa cells [37]. Another in vitro experiment of the antiproliferative effects of PAC-rich fractions from blueberry fruits on PCa cells was also conducted. PCa cell proliferation was markedly suppressed upon treatment with 20 μg/mL of PACs fractions four and five, with high degrees of polymerization in wild (DPn = 4.8 and 5.3) and cultivated (DPn = 3.1 and 4.6) blueberries [38]. The methanolic extracts from blueberries (ACNs, PACs, Qu, and Km) were tested against PCa cells at concentrations ranging from 25 to 200 μg/mL. The results showed antiproliferative effects on PCa cells upon exposure to increasing concentrations of berry extracts [39]. 

An experimental model assessed the in vitro anticarcinogenic activity of two blueberry extracts (Tifblue and Premier) in CC cells. The extracts showed significant inhibition of mutagenesis caused by the metabolically and directly acting activated carcinogen methyl methanesulfonate [40]. The combinational treatment of CC cells with blueberry extracts and radiotherapy was shown to inhibit proliferation while activating apoptosis-related gene expression in vitro [41]. ACNs, anthocyanidin, and proanthocyanidin extracts from lowbush blueberries have been shown to suppress the proliferation and viability of CC cells in vitro through mechanisms involved in apoptosis induction and cell cycle arrest in both S and G_2_/M phases at concentrations of 100–600 mg mL^−1^. In addition, ACNs at the highest concentration of 600 mg mL^−1^ exhibit low cytotoxic effects on CC cells compared to other extracts [42]. The antiproliferative effects of ACNs from Gardenblue blueberries have been confirmed in a recent in vitro experiment. The results showed that ACNs in combination with chemotherapeutic drugs (cisplatin and doxorubicin) inhibit the growth of CC cells at a concentration of 51.98 μg/mL; however, the mechanisms behind this process have not been documented [43]. A significant inhibition of OC and CC cell proliferation in a dose-dependent manner was reported in in vitro experiments following treatment with blueberry juice. However, the exact mechanisms underlying the antiproliferative role of blueberries have not been determined [44]. It has been shown that blueberry juice, at a concentration of 16 mg/mL, enhanced antiproliferative effects in nude mice through downregulation of COX-1 and COX-2 expression in OC cells [45]. 

The main results related to the therapeutic role of blueberry bioactives in RCs are presented in Table 1. 

## 5. The Therapeutic Role of Cranberry Bioactives in Reproductive Cancers

Evidence from experimental studies and randomized controlled trials (RCTs) suggests that cranberries and/or their bioactives may have therapeutic potential against RCs.

### 5.1. Experimental Studies

A limited number of experiments have revealed that cranberry bioactives exert anti-tumour, antiproliferative, anti-viability, anti-metastatic, anti-invasive, anti-angiogenic, apoptotic, and autophagic effects in RC cells, particularly PCa, BC, and OC cells. Cranberry bioactives have demonstrated antiproliferative and tumour growth inhibition activities in PCa cells, but the mechanisms underlying these activities have not been identified. Treatment with a fraction of cranberry press cake extract containing 10 and 250 mg/mL of flavonoids (ACNs, flavonols, PACs, and epicatechin) resulted in a reduction in PCa cell proliferation in vitro [46]. In another in vitro experiment, higher concentrations (in particular 200 μg/mL) of total cranberry extract and its isolated fractions enriched in flavonoids (ACNs, PACs, and epicatechin) were shown to inhibit PCa cell proliferation [47]. The PACs-rich fraction isolated from cranberry press cake showed significant suppression of tumour growth in PCa when injected in mice at a dose of 100 mg/kg [48].

An in vitro experiment showed that 25 μg/mL of cranberry PACs repressed PCa cell viability through the inhibition and/or activation of multiple MMP regulators, with no cytotoxicity observed [49]. Exposure of PCa cells to whole cranberry extracts (ACNs, Qu, and PACs) was studied in vitro and revealed cytotoxic effects on cell viability at various concentrations (10, 25, and 50 μg/mL^−1^), resulting in cell cycle arrest in the G_1_ phase [50]. An in vitro investigation of the effects of flavonol and PACs-enriched fractions of cranberry on PCa cells showed apoptotic effects against the DU145 cell line at different concentrations (25, 50, and 100 μg/mL) by activating the expression of genes responsible for cytochrome C release from the mitochondria, including caspase-8/9, prostate apoptosis response-4 gene (Par-4), truncated Bid (tBid), and Bcl-2-associated X protein (Bax) [51]. It was demonstrated in vitro that 1 μg/mL^−1^ and 10 μg/mL^−1^ of ursolic acid and its *cis* and *trans*-3-O-*p*-hydroxycinnamoyl esters extracted from three cranberry species (*V. angustifolium*, *V. vitis-idaea*, and *V. oxycoccus*) inhibit tumour growth, invasion, and metastasis of PCa cells by reducing the expression of MMPs [52]. 

An in vivo experiment in rats demonstrated that treatment with cranberry juice concentrate extracted with ethyl acetate at doses of 1.0 and/or 0.5 mL/day over six weeks resulted in a significant decrease in the number and size of urinary BC tumours. Upon treatment, ACNs and PACs were detected in the serum and urine samples, while no Qu was observed, suggesting its low bioavailability [53]. The anticancer effects of cranberry-derived flavonoids were evaluated in an in vivo model of BC. The results revealed tumour growth inhibitory and antiproliferative effects of Qu 3-O-glucoside, aglycone Qu, and Myr against BC cells at a high concentration of 200 μM [54].

The effects of cranberry PACs in combination with paraplatin on platinum-resistant OC cells have been investigated in vitro. The combinational treatment resulted in the inhibition of tumour cell proliferation and viability, along with the induction of apoptosis in SKOV-3 cells at concentrations ranging from 0–150 μg/mL. Cranberry PAC pretreatment also increases paraplatin cytotoxicity in SKOV-3 cells [55]. An in vitro experiment has reported that cranberry PACs, when used at concentrations ranging from 25–100 μg/mL in OC cells, inhibit cell proliferation, viability, and angiogenesis and induce cytotoxicity, apoptosis, and cell cycle arrest in the G_2_/M phase. Cranberry PAC treatment increases intracellular reactive oxygen species (ROS) production associated with cytotoxicity, activates apoptotic markers, and inhibits vascular endothelial growth factor (VEGF) in OC cells [56]. Treatment of OC cells with various concentrations (12.5–200 μg/mL) of cranberry Qu aglycone and PACs in vitro inhibited viability, induced apoptosis, and arrested the cell cycle in the G_2_/M, S/G_2_, and G_1_/S phases. The mechanisms underlying these effects are associated with the activation of genes involved in cell apoptosis and the downregulation and/or upregulation of genes associated with cell cycle and viability. The treatment also resulted in potentiated cisplatin cytotoxicity at low concentrations of 15 and 60 μg/mL [57]. 

Findings from in vitro and/or in vivo studies evaluating the therapeutic roles of cranberry bioactives in RCs are summarized in Table 2. 

### 5.2. Clinical Trials

A few RCTs have shown contradictory results when evaluating the effectiveness of cranberry in reducing the incidence of urinary tract infections (UTIs)/urinary symptoms in RC patients undergoing radiation treatment. One trial showed no differences in urinary symptoms in PCa patients consuming cranberry or apple juice during radiation treatment [58]. Another trial revealed that cranberry capsules have higher antioxidant and/or anti-inflammatory activities (assessed as the suppression of neutrophil superoxide production) than beetroot capsules. However, PCa patients who received cranberry capsules had severe radiation cystitis symptoms compared with those who received beetroot capsules [59]. Analysis of BC and CC patients who received a placebo beverage compared with those who received cranberry juice showed that grade 3 urinary symptoms and UTIs significantly increased [60]. 

Administration of cranberry capsules containing PACs to PCa patients led to a significant decrease in radiation cystitis symptoms (less pain/burning upon urination, blood in urine, and leaking/dribbling) compared with the administration of placebo capsules [61]. A trial reported that a daily intake of 200 mg of highly standardized cranberry extract for 6–7 weeks reduced urinary discomfort (urinary frequency and nocturia) and days of treatment with inflammatory drugs/antibiotics in PCa patients [62]. Results from a trial demonstrated that the serum prostate-specific antigen and urine, blood, or prostate tissue markers were reduced in PCa patients prior to radical prostatectomy when treated with cranberry fruit powder compared to placebo [63]. 

Table 3 lists findings from RCTs evaluating the role of cranberry in RC treatment.

## 6. The Therapeutic Role of Bilberry and Lingonberry Bioactives in Reproductive Cancers

There is limited experimental evidence suggesting that bilberry bioactives exert anti-tumour growth, antiproliferative, and/or apoptotic effects on PCa and OC cells. Additionally, the mechanisms involved in these effects have not been fully explored. Methanolic extract from *V. myrtillus*. L was shown to be effective at inducing apoptosis while inhibiting PCa cell growth and proliferation under hypoxic conditions at different dilutions [64]. One in vitro and in vivo experiment reported antiproliferative and cell growth inhibition effects of bilberry anthocyanidin aglycone (Anthos) and exosomal Anthos (ExoAnthos) on PCa and OC cells at different concentrations (7, 9, 10, 111, 112, and 357 µM), while no Anthos and ExoAnthos toxicities were observed [65]. Another in vitro and in vivo experiment demonstrated that bilberry Anthos and ExoAnthos led to the inhibition of proliferation/tumour growth of cisplatin-resistant and drug-sensitive OC cells. Anthos exerted antiproliferative effects on OC cells at a concentration of 75 μM when combined with cisplatin. The combination of Anthos with paclitaxel chemotherapy reduced the expression of *p*-glycoproteins (PgP) in OC cells, while treatment with the Exopaclitaxel formulation resulted in tumour growth inhibition, suggesting that the formulation may improve oral Anthos bioavailability in mice [66]. The lingonberry extracts (cyanidin and procyanidins) were shown to inhibit in vitro CC cell proliferation and viability at concentrations ranging from 25 to 75 μg/mL [67]. 

Table 4 summarizes findings from experimental studies that have evaluated the therapeutic roles of bilberry and lingonberry bioactives in RCs.

## 7. Limitations

The results revealed that *Vaccinium* bioactives exert anticancer effects in different types of RCs, which have mostly been evaluated in in vitro experimental models. Cranberry has been evaluated for its clinical relevance, with the focus being only on assessing its effectiveness in reducing UTIs/urinary symptoms. Only the effects of PACs as bioactive compounds have been reported. Additionally, current trials evaluating anticancer effects have not predicted the clinical benefits and safety considerations of cranberry in cancer patients. As clinical trials were randomised, the total number of cancer patients was unequally distributed between the cranberry group and the placebo group. 

The bioactive compounds were not identified in a few experiments evaluating the anticancer effects of blueberries in PCa, CC, and OC cells. The mechanisms through which *Vaccinium* bioactives were shown to exert anticancer effects in RC cells have not been fully explored. Only a few experiments showed that the anticancer mechanisms of blueberry and/or cranberry bioactives in RC cells are controlled via multiple genes without altering the cellular signalling pathways. 

While a few in vivo experiments showed therapeutic success when testing *Vaccinium* bioactives against PCa, BC, and OC cells in mouse models, it is unlikely to succeed in translation to clinical trials since human metastases or tumours are not accessible for direct injection of therapies. *Vaccinium* bioactives have been inadequately examined for their bioavailability in a few in vivo experiments, which indicates a challenge in evaluating their potential therapeutic effects in RC cells. There are no in vivo experiments demonstrated that *Vaccinium* bioactives enhance the cytotoxicity of chemotherapeutic drugs in RC cells. Although experimental models showed effective results of *Vaccinium* bioactives in RC treatment, the optimal concentration has not been clearly determined. A limited number of experiments showed high cytotoxicity of *Vaccinium* bioactives on RC cells, which could be helpful in the inhibition of tumour growth by enhancing apoptosis and cell cycle arrest. 

## 8. Conclusions and Future Directions

*Vaccinium* berries include various species of fleshy berry crops that contain high levels of bioactive compounds with anticancer effects. The results from experimental models indicate that *Vaccinium* bioactives may have therapeutic potential against RC cells through the inhibition and/or activation of RC-mediated molecular genes. ACNs, PACs, flavonols, flavanols, ursolic acid, and phenolic acids are the most bioactive compounds extracted from blueberries, cranberries, bilberries, and lingonberries that have exhibited a variety of effects against RC cells, including antiproliferative, anti-tumour growth, anti-viability, anti-invasion, anti-adhesion, anti-metastasis, anti-angiogenic, anti-inflammatory, apoptotic, and autophagic activities. Treatment with ACNs/PACs extracted from blueberry, cranberry, and bilberry in combination with chemotherapy and/or radiotherapy demonstrated inhibition of proliferation, viability, and tumour growth, along with induction of apoptosis in CC and OC cells. A limited number of RCTs of treatment with cranberry showed a significant reduction in UTIs/urinary symptoms in PCa, BC, and CC patients.

The translation of experimental models into clinical trials is rare and success is not guaranteed. In the experimental models, there is a need to use an accurate tumour model and dosage of *Vaccinium* bioactives in RC treatment to enhance the clinical success rate. It is also important to include the route of *Vaccinium* bioactives administration and appropriate tumour controls to ensure that the treatment does not lead to any clinical side effects. Therefore, more experimental models are needed to verify their treatment effectiveness before translation to human clinical trials. In vivo experimental models are also needed to evaluate the therapeutic effects and safety of *Vaccinium* bioactives in RCs.

The potential mechanisms of *Vaccinium* bioactives in RC treatment need to be further elucidated in in vivo/vitro experimental models. Additionally, more experimental models focusing on optimizing the bioavailability of *Vaccinium* bioactives are needed to evaluate their therapeutic value in RCs. Further experimental and clinical trials are required to validate the anticancer effects of *Vaccinium* bioactives in RC cells when used in combination with chemotherapy and/or radiotherapy. Additional clinical studies are required to test the toxicity/safety of *Vaccinium* bioactives in RC patients. There is also still a need for more clinical trials of the medicinal properties of *Vaccinium* bioactives with multiple therapeutic effects in RCs. 

## Figures and Tables

**Table 1:** Therapeutic roles of blueberry bioactives in RCs.

Cancer Models	Cancer Types	Cell Lines	Bioactive Extracts	Treatment	Therapeutic Role	Mechanisms of Action	Ref.
In vitro	PCa	PC-3	NA	PC-3 cells were treated with 0, 10, 20, 30, 40, and 50 μL/mL of three different blueberries and incubated for 48 h	Inhibition of cell proliferation and inflammation Induction of cell cycle arrest	cdk4/6, cyclin D1/D3, TNFα, COX-2, NFκB ↓	[34]
In vitro	PCa	DU145	ACNs (cyanidin, peonidin, petunidin, delphinidin, and malvidin), flavonol (Qu), PACs	DU145 cells were treated with 0.1, 0.5, and 1.0 mg/mL fractions of lowbush blueberries and incubated for 24 h	Inhibition of metastasis formation Induction of cell apoptosis	caspase-3 ↑, MMP-2/9 ↓	[35]
In vitro	PCa	DU145	ACNs (cyanidin, peonidin, petunidin, delphinidin, and malvidin), flavonol (Qu), PACs	DU145 cells were treated with 0.1, 0.5, and 1.0 mg/mL fractions of lowbush blueberries and incubated for 24 h	Inhibition of metastasis formation	TIMP-1/2 ↑, MMP-2/9, PKA, MAP ↓	[36]
In vitro	PCa	LNCaP	PACs	LNCaP cells were treated with 5, 10, 12, 15, 20, 30, and 40 μg/mL of PAC-rich fractions of wild blueberry and 2.38 mM QU standard and incubated for 24 h	Inhibition of cell proliferation and adhesion	NA	[37]
In vitro	PCa	LNCaP, DU145	PACs	PCa cells were treated with 0, 20, 40, 60, 80, 100, and 120 μg/mL of PAC-rich fractions of two blueberry fruits and 2.38 mM QU standard and incubated for 24 h	Inhibition of cell proliferation	NA	[38]
In vitro	PCa	LNCaP	ACNs (cyanidin, peonidin, petunidin, delphinidin, and malvidin), flavonol (QU, Km), PACs	LNCaP cells were treated with blueberry extracts ranging from 25 to 200 μg/mL in concentration and incubated for 48 h	Inhibition of cell proliferation	NA	[39]
In vitro	CC	CaSki, SiHa	NA	CC cells were treated with different concentrations of four extracts (ethyl acetate, hexane/ethyl acetate, acetone/water, and ethanol) of Tifblue and Premier blueberries and incubated for 48 h.	Inhibition of mutagenesis	Carcinogen formation by methyl methanesulfonate ↓	[40]
In vitro	CC	SiHa	NA	SiHa cells were treated with 50 mg/mL blueberry extracts for 24 h, followed by radiotherapy at 4 Gy for 48 h	Inhibition of cell proliferation Induction of cell apoptosis	caspase-3, TUNEL+ cells, TRAIL ↑, cyclin D/E ↓	[41]
In vitro	CC	HeLa	ACNs (cyanidin, peonidin, petunidin, delphinidin, and malvidin), anthocyanidin, proanthocyanidin	HeLa cells were treated with 0, 100, 200, 400, and 600 μg/mL^−1^ of lowbush blueberry extracts and incubated for 24 h	Inhibition of cell proliferation and viability Induction of apoptosis and cell cycle arrest	caspase-3, p53, p38 MAPK ↑, cyclin D1 ↓	[42]
In vitro	CC	HeLa	ACNs (cyanidin, peonidin, petunidin, delphinidin, and malvidin)	HeLa cells were treated with 52 μg/mL of Gardenblue blueberry ACNs and chemotherapeutic drugs (cisplatin and doxorubicin)	Inhibition of cell proliferation	NA	[43]
In vitro	OC and CC	A2780 (OC), HeLa (CC)	ACNs (cyanidin, peonidin, petunidin, delphinidin, and malvidin)	Cells were treated with 0, 50, 100, 150, 200, 250, 300, 350, 400, 450, and 500 μg/mL of ACN-rich fractions of blueberry juice and incubated for 24 h	Inhibition of cell proliferation and viability	NA	[44]
In vivo	OC	SKOV3	NA	BALB/c mice were fed daily with 100, 200, and 400 mg/kg of blueberry juice. SKOV3 cells were treated with 0, 1, 2, 4, 8, or 16 mg/mL of blueberry juice and incubated for 24, 48, or 72 h	Inhibition of cell proliferation	COX-1/2 ↓	[45]

(↓) Decrease; (↑) increase; NA = not available.

**Table 2:** Therapeutic roles of cranberry bioactives in RCs.

Cancer Models	Cancer Types	Cell Lines	Bioactive Extracts	Treatment	Therapeutic Role	Mechanisms of Action	Ref.
In vitro	PCa	LNCaP, DU145	ACNs, flavonols, PACs, flavan-3-ols (epicatechin)	PCa cells were treated with fractions of cranberry press cake extract (0, 100, 200, 300, 400, 500, and 600 mg/mL) and incubated for 4d	Inhibition of cell proliferation	NA	[46]
In vitro	PCa	RWPE-1, RWPE-2, 22Rv1	ACNs (cyanidin, peonidin), PACs, total polyphenols	PCa cells were treated with fractions of total cranberry extract (50, 100, and 200 μg/mL) and incubated for 48 h	Inhibition of cell proliferation	NA	[47]
In vivo	PCa	DU145	PACs	Female Balb/c mice were injected with 100 mg/kg PACs 3, 5, 7, 10, 12, 14, 17, 19, and 21 days after tumour implant.	Inhibition of tumour growth	NA	[48]
In vitro	PCa	DU145	PACs	DU145 cells were treated with 0–25 μg/mL of cranberry PACs and incubated for 24 h	Inhibition of cell viability	TIMP-2, p38, ERK 1/2, c-jun ↑, MMP-2/9, EMMPRIN, PI3K, Akt, NFkB p65, c-fos ↓	[49]
In vitro	PCa	DU145	ACNs (cyanidin, peonidin), flavonols (Qu), PACs	DU145 cells were treated with whole cranberry extracts (10, 25, and 50 μg/mL^−1^) and incubated for 24 h	Inhibition of cell viability Induction of cell cycle arrest	p27 ↑, CDK4, cyclin A/B1/D1/E ↓	[50]
In vitro	PCa	DU145	ACNs (cyanidin, peonidin), flavonols (Qu, Myr), PACs	DU145 cells were treated with flavonol and PAC-enriched fractions of cranberry (10, 25, 5, and 100 μg/mL) and incubated for 24 h	Induction of cell apoptosis	caspase-8/9, par-4, cytochrome-C, tBid, Bax ↑	[51]
In vitro	PCa	DU145	Ursolic acid	DU145 cells were treated with 1 μg/mL^−1^ and 10 μg/mL^−1^ of ursolic acid and its *cis* and *trans*-3-O-*p*-hydroxycinnamoyl esters extracted from cranberries and incubated for 24 h	Inhibition of tumour growth, invasion, and metastasis	MMP-2/9 ↓	[52]
In vivo	BC	NA	NA	Female Fischer rats were injected with 1.0 or 0.5 mL/day of cranberry juice concentrate for a period of six weeks	Inhibition of tumour growth and cell proliferation	NA	[53]
In vivo	BC	RT4, SCABER, SW-780	Flavonols (Qu, aglycone Qu, Myr)	BC cells were treated with cranberry-derived flavonoid concentrations ranging from 0.3–200 μM and incubated for 72 h	Inhibition of tumour growth and cell proliferation	NA	[54]
In vitro	OC	SKOV-3	PACs	SKOV-3 cells were treated with cranberry PAC fractions (concentrations ranging from 0–150 μg/mL) and 4.5 μg/mL paraplatin and incubated for 48 h	Inhibition of cell proliferation and viability Induction of cell apoptosis	NA	[55]
In vitro	OC	SKOV-3	PACs	SKOV-3 cells were treated with cranberry PACs (concentrations ranging from 12.5–100 μg/mL) and incubated for 24 h	Inhibition of cell proliferation, viability, and angiogenesis Induction of cell cycle arrest and apoptosis	ROS, caspase-3/7/8 ↑, Akt, PARP, VEGF ↓	[56]
In vitro	OC	SKOV-3, OVCAR-8	PACs, Qu aglycone	OC cells were treated with cranberry PACs and Qu aglycone concentrations ranging from 0–200 μg/mL and incubated for 24–48 h	Inhibition of cell viability Induction of cell cycle arrest and apoptosis	caspase-3, p21, p27, CDK-2 ↑, PARP, EGFR, MAPK, ERK, RAF, cyclin D1, phospho-histone H3, DNA-PK ↓	[57]

(↓) Decrease; (↑) increase; NA = not available.

**Table 3:** Summary of clinical trials of cranberry in RC patients.

Study Characteristics	Study Focus	Intervention	Effects	Ref.
Total subjects = 112 PCa patients (cranberry group = 55, apple group = 57)	Urinary symptoms	Patients consumed 354 mL of cranberry juice or apple juice a day over two weeks during radiation treatment	No significant effects on urinary symptoms were observed	[58]
Total subjects = 101 PCa patients (cranberry capsules group = 51, placebo = 50)	Cystitis symptoms	Patients received two capsules containing cranberry PACs or a beetroot-containing placebo twice a day during radiation treatment and two weeks after	Patients in the cranberry arm developed worse radiation cystitis symptoms compared with those in the placebo arm	[59]
Total subjects = 128 BC and CC patients (cranberry group = 64, placebo = 64)	Urinary symptoms, UTIs	Patients received ≥16,000 mL of cranberry juice or a placebo beverage twice a day over four weeks during radiation treatment and two weeks after	Patients in the placebo arm experienced more urinary symptoms and UTIs during and after radiation treatment compared with those in the treatment arm	[60]
Total subjects = 40 PCa patients (cranberry capsules group = 20, placebo = 20)	Cystitis symptoms	Patients received one capsule containing cranberry PACs or a magnesium stearate, gelatin, colloidal silica, and gelatin-containing placebo once a day during radiation treatment and two weeks after	Patients in the cranberry arm had fewer severe cystitis symptoms compared with those in the placebo arm during and after radiation treatment	[61]
Total subjects = 924 PCa patients (enteric-coated, highly standardized cranberry group = 489, untreated group = 435)	UTIs	Patients treated with one tablet/day of 200 mg of highly standardized cranberry extract over 6–7 weeks of radiation treatment	Patients demonstrated a significant reduction in urinary discomfort and the use of antibiotics/anti-inflammatory drugs	[62]
Total subjects = 64 PCa patients (cranberry group = 32, placebo = 32)	Serum prostate-specific antigen; blood, urine, and prostate tissue markers	Patients received 1500 mg of cranberry fruit powder daily for one month, or one capsule containing 500 mg of canola oil, Blue 1 Lake, sodium aluminium silicate, STAR-DRI^®^ 1015A (Tate & Lyle Solutions, Sycamore, IL, USA) maltodextrin (placebo) 21 days before surgery	Patients in the cranberry arm showed a significant reduction in serum prostate-specific antigen, serum gamma-glutamyltranspeptidase, and urinary beta-microseminoprotein, along with upregulated insulin-like growth factor-1 compared with those in the placebo arm	[63]

**Table 4:** Therapeutic roles of bilberry and lingonberry bioactives in RCs.

Cancer Models	Cancer Types	Cell Lines	*Vaccinium* Species	Bioactive Extracts	Treatment	Therapeutic Role	Mechanisms of Action	Ref.
In vitro	PCa	LNCaP, DU145, PC3	Bilberry	ACNs (cyanidin, peonidin, petunidin, delphinidin, and malvidin), flavonol (Qu and Myr), flavanol (epicatechin), phenolic acids (Gallic, p-Coumaric, caffeic, and chlorogenic acids)	PCa cells were treated with bilberry extract and a recombined standard mixture at distinct dilutions (1/100 *v*/*v* dilution, (1/400 *v*/*v*, and 1/800 *v*/*v* dilution), and incubated for 7d	Inhibition of tumour growth and cell proliferation Induction of cell apoptosis	Hypoxia ↑	[64]
In vitro/vivo	PCa and OC	DU145, PC3 (PCa); OVCA432 (OC)	Bilberry	Anthos	Mice were randomized into vehicle (PBS), ExoAnthos (5 mg Anthos and 50 mg Exo protein/kg b.wt.), and Anthos (10 mg/kg b. wt.) groups PCa and OC cells were treated with various concentrations of ExoAnthos, Anthos, or exosomes and incubated for 72 h	Inhibition of tumour growth and cell proliferation	NA	[65]
In vitro/vivo	OC	A2780, A2780/CP70, OVCA432, OVCA433	Bilberry	Anthos	Mice were treated, through oral gavage, with Anthos (6 and 30 mg kg^−1^ b. wt) and exosomal formula (mg kg^−1^ Anthos and 60 mg kg^−1^ Exo) (study 1); Mice were also treated with paclitaxel (4 mg kg^−1^ b. wt) and exosomal formula of paclitaxel (4 mg PAC kg^−1^ b. wt and 60 mg Exo kg^−1^ b. wt) OC cells were treated with different concentrations (0, 50 100, 150, 200, and 300 µM) of Anthos and paclitaxel and incubated for 72 h	Inhibition of tumour growth and cell proliferation	PgP ↓	[66]
In vitro	CC	HeLa	Lingonberry	ACNs (cyanidin), tannin (procyanidins)	HeLa cells were treated with fractions of lingonberry extract (concentrations ranging from 25–75 μg/mL) and incubated for 72 h	Inhibition of cell proliferation and viability	NA	[67]

(↓) Decrease, (↑) increase; NA = not available.

## Data Availability

Not applicable.

## Abbreviations

ABA	Abscisic acid
ACNs	Anthocyanins
Akt	Threonine kinase
Anthos	Anthocyanidin aglycone
AR	Androgen receptor
ARID1A	AT-rich interaction domain 1A
Bax	Bcl-2-associated X protein
BC	Bladder cancer
CC	Cervical cancer
CDK	Cyclin-dependent kinase
COX-2	Cyclooxygenase-2
DNA-PK	DNA-dependent protein kinase
DPn	Polymerization
EGFR	Epidermal growth factor receptor
EMMPRIN	Extracellular matrix metalloproteinase inducer
ERK	Extracellular-signal regulated kinase
ExoAnthos	Exosomal Anthos
FGFR3	Fibroblast growth factor receptor 3
FOXA1	Forkhead box A1
HIF1α	Hypoxia inducible factor-1α
Iso	Isorhamnetin
KDM6	Lysine (K)-specific demethylase 6
Km	Kaempferol
KRAS	Kirsten rat sarcoma viral oncogene homologue
MAP	Mitogen-activated protein
MMPs	Matrix metalloproteinases
Myr	Myricetin
NF_k_B	Nuclear factor kappa-B
OC	Ovarian cancer
p21	Protein 21
p27	Protein 27
p38 MAPK	Protein 38 mitogen-activated protein kinases
p53	Protein 53
PACs	Proanthocyanidins
PAM50	50-gene signature
Par-4	Prostate apoptosis response-4 gene
PARP	Poly(ADP-ribose) polymerase
PCa	Prostate cancer
PCNA	Proliferating cell nuclear antigen
PCS	Prostate cancer 14-pathway
PgP	*p*-glycoproteins
PI3K	Phosphoinositide 3-kinase
PIK3CA	P110alpha catalytic subunit of PI3K
PKA	Protein kinase A
PPARγ	Peroxisome proliferator-activated receptor gamma
PR	Progesterone receptor
PTEN	Phosphatase and tensin homolog
Qu	Quercetin
RAF	Retinoblastoma tumour suppressor protein-proto-oncogene
RB1	Retinoblastoma gene 1
RCs	Reproductive cancers
RCTs	Randomized controlled trials
ROS	Intracellular reactive oxygen species
SCCC	Small-cell carcinoma of the cervix
STAT3	Signal transducer and activator of transcription3
tBid	Truncated Bid
TCGA	Cancer genome atlas
TIMPs	Metalloproteinases
TNF-α	Tumour necrosis factor
TP53	p53 tumour-suppressor gene
TRAIL	Tumour necrosis factor-related apoptosis-induced ligand
TUNEL+	TUNEL-positive tumour cells
UTIs	Urinary tract infections
VEGF	Vascular endothelial growth factor
WT1	Wilms’ tumour gene 1
